# Antibiotic resistance and typing of *agr* locus in *Staphylococcus aureus* isolated from clinical samples in Sanandaj, Western Iran

**DOI:** 10.22038/ijbms.2020.46064.10661

**Published:** 2020-10

**Authors:** Samira Saedi, Safoura Derakhshan, Ebrahim Ghaderi

**Affiliations:** 1 Student Research Committee, Kurdistan University of Medical Sciences, Sanandaj, Iran; 2 Liver and Digestive Research Center, Research Institute for Health Development, Kurdistan University of Medical Sciences, Sanandaj, Iran; 3 Zoonoses Research Center, Research Institute for Health Development, Kurdistan University of Medical Sciences, Sanandaj, Iran

**Keywords:** Agr protein, Drug resistance, Iran, Methicillin, Staphylococcus aureus

## Abstract

**Objective(s):**

Infections by *Staphylococcus aureus *remain an important health problem. The aims were to detect *mecA*, staphylococcal cassette chromosome mec (SCCmec), *accessory gene regulator (agr)*, and integrons in *S. aureus *and to investigate the relationship of *agr* types with antibiotic resistance of isolates.

**Materials and Methods:**

In this cross-sectional study, 70 *S. aureus* isolates were collected between December 2017 and May 2018 from clinical specimens of patients in two hospitals of Sanandaj, western Iran. Susceptibility was determined by disk diffusion for 9 antibiotics and by vancomycin E test. The *mecA*, classes 1-3 integrons, SCC*mec* I-V, and *agr* I-IV were detected by polymerase chain reaction. A *P*-value<0.05 was considered significant.

**Results:**

The most effective antibiotics were linezolid, vancomycin, and trimethoprim-sulfamethoxazole (above 90% sensitivity). Of the 70 isolates, 17.1% were methicillin-resistant *S. aureus *(MRSA), 8.6% carried class 1 integron, 11.4% carried *mecA*, 17.1% carried *agr* I, and 30% carried *agr* III. SCC*mec* III and SCC*mec*V were detected. An association was found between resistance to certain antibiotics and the presence of *agr* I (*P*-value<0.05). Conversely, the prevalence of *agr* III in susceptible strains was higher than non-susceptible strains, and no MRSA isolates belonged to *agr* III (*P*-value<0.05).

**Conclusion:**

These data suggest that *agr* activity may influence the resistance of *S. aureus* to antibiotics. Although the prevalence of *mecA* and integron was relatively low, the identification of such strains calls for serious health concerns; thus highlights the need to monitor drug resistance in *S. aureus*.

## Introduction

*Staphylococcus aureus* is one of the leading causes of bacterial infections in humans and is responsible for a wide range of diseases including septicemia, meningitis, endocarditis, osteomyelitis, septic arthritis, toxic shock syndrome and, food poisoning. 

*S. aureus* has become one of the most important antibiotic-resistant pathogens in hospitals and communities, worldwide (1). In the 1960s, methicillin was used to treat infections caused by *S. aureus*, but after a short period, methicillin-resistant *S. aureus* (MRSA) strains emerged (2). MRSA was first recognized as being acquired from the hospital settings (hospital-acquired, HA-MRSA), but the onset of MRSA infection in the community (community-acquired, CA-MRSA), has been described with increasing frequency (1).

Methicillin resistance in *S. aureus *is mainly mediated by an acquired penicillin-binding protein (PBP), named PBP2A which has a low affinity for beta-lactam antibiotics. PBP2A is encoded by the *mecA *gene, which is located on the *S. aureus *chromosome as part of a large mobile genetic element called staphylococcal cassette chromosome *mec*, or SCC*mec* (3). SCC*mec* consists of two main components: the *ccr* gene complex, which causes the mobility of SCC*mec*, and the *mec* gene complex. Various types of SCC*mec* have been identified, but the more dominant types are SCC*mec *I-V (4). SCC*mec *types I, IV, and V cause resistance to beta-lactam antibiotics, but types II and III determine resistance to multiple antibiotics. Methicillin-resistant strains of *S. aureus* are clinically important and have gained the attention of researchers because a single genetic element confers resistance to beta-lactam antibiotics (1). 

The expression of many virulence genes in *S. aureus *is regulated by the accessory gene regulator (*agr*) system which responds to cell density-dependent stimuli. At high cell densities, this quorum-sensing system downregulates the expression of surface virulence factors, while up-regulating the expression of secreted virulence factors. The activity of the *agr* system involves two transcripts, RNAII and RNAIII, where RNAII encodes four proteins that generate the *agr*-sensing mechanism and as a result of their activation, RNAIII (the effector molecule of the *agr *locus) is produced and controls the expression of a large number of virulence genes (5). There are four types of *agr *systems, referred to as *agr* I through *agr *IV. Under certain conditions, *agr* activity may influence the type of infection and resistance of *S. aureus* to antibiotics. For example, the association of *agr* IV with the presence of exfoliative toxin genes and a link between the* agr *types I and II with intermediate vancomycin resistance have been previously reported (6). However, the role of *agr *in human infections and particularly in antibiotic treatment is controversial (7). 

The crucial role of integrons in the transfer of antibiotic resistance has been demonstrated. Integrons, the unit of genes, located in bacterial chromosome, plasmid or transposon, have the capacity to transfer antibiotic resistance genes. An integron consists of three elements: a gene (*intI*) that encodes integrase, a recombination site (*attI)*, and a promoter gene (8). Among different classes of integrons, classes 1*, *2, and 3 are most commonly associated with the spread of antibiotic resistance genes. Although the role of integrons in the spread of antibiotic resistance genes among Gram-negative bacteria has been well studied (9), less is known about the prevalence of integrons in Gram-positive bacteria, especially in *S. aureus.*

Considering the importance of *S. aureus *strains in the hospital and the community, this study was conducted to detect *mecA*, SCC*mec *types I-V*, agr *types I-IV and classes 1-3 integrons in *S. aureus* isolated from different clinical specimens in Sanandaj city, western Iran and to investigate the association of *agr *types with antibiotic resistance of isolates.

## Materials and Methods

***Bacterial isolates and identification***

A total of 70 non-duplicated *S. aureus* were isolated between December 2017 and May 2018 from different clinical specimens of patients in two teaching general hospitals affiliated to Kurdistan University of Medical Sciences in Sanandaj, Iran. Sanandaj is the capital of Kurdistan Province in western Iran. All isolates were identified by the standard biochemical tests for *S. aureus *such as gram stain, catalase, coagulase, DNase, and fermentation of mannitol (10). 

A polymerase chain reaction (PCR) method was used to confirm the identification of *S. aureus*, using primers for *nuc *gene (encoding a thermostable nuclease); nuc1: 5′- GCGATTGATGGTGATACGGTT -3′ and nuc2: 5′- AGCCAAGCCTTGACGAACTAAAGC -3′ (amplicon size: 279 bp) (11). PCR conditions were as follows: initial denaturation at 94 ^°^C for 5 min; followed by 35 cycles of 94 ^°^C for 1 min, 61 ^°^C for 1 min, and 72 ^°^C for 1 min. The final extension was at 72 ^°^C for 5 min. *S. aureus *ATCC25923 was used as a positive control. The isolates were stored at -70 ^°^C in Trypticase soy broth (Q- Lab, US), containing 15% v/v glycerol for further investigations.

***Antibiotic susceptibility test***

Antimicrobial susceptibility testing was performed by the disk diffusion method according to the 2019 Clinical and Laboratory Standards Institute (CLSI) guidelines (12) with the following 9 antibiotic disks (Rosco, Denmark): cefoxitin (30 µg, for detection of MRSA), penicillin (10 units), erythromycin (15 µg), clindamycin (2 µg), gentamicin (10 µg), linezolid (30 µg), tetracycline (30 µg), trimethoprim-sulfamethoxazole (1.25-23.75), and ciprofloxacin (5 µg). Furthermore, the susceptibility of isolates to vancomycin was determined by the E. test (bioMerieux, France). Briefly, Mueller-Hinton agar (Q-Lab, US) plates were inoculated with the broth suspensions equivalent to 0.5 McFarland of each isolate. Disks and E. test strips were placed onto the plates and incubated at 35 ^°^C for 16–18 hr (24 hr for vancomycin). The inhibition zones were measured and interpreted according to the 2019 CLSI guidelines (12).* S. aureus* ATCC25923 and *S. aureus* ATCC29213 strains were used as the quality controls.

***Genomic DNA extraction***

Genomic DNA was extracted by the freeze-thaw method (13) and used as the template for PCR reactions. The suspensions of each isolate in Tris-EDTA (TE) buffer were boiled at 100 ^°^C for 10 min and immediately placed on ice for 5 min. After three cycles of freezing-thawing, the tubes were centrifuged and the supernatants containing DNA were removed and stored at -20 ^°^C. The quantity of each DNA extract was determined by measuring the absorbance at 260 nm to estimate the DNA concentration and by calculating the ratio of A260/A280 to determine purity. DNA samples within the range of 1.6–2 were considered pure (14).

***Detection of genes***

PCR assay was used to detect *mecA*, SCC*mec *types I-V*, agr *types I-IV, and classes 1-3 integrons by specific primers (Table 1). The reactions were done in a volume of 25 µl containing 1.5 mM MgCl_2_, 0.2 mM of each dNTP, 1 U Taq polymerase, 1X reaction buffer, 0.4 µM of each primer (SinaClon, Iran), and 3 µl DNA template. PCR was performed in a thermal cycler (Eppendorf, Germany) under the following conditions: initial denaturation at 94 ^°^C for 5 min, then 35 cycles of denaturation at 94 ^°^C for 1 min, annealing at different temperatures (Table 1) for 1 min, extension at 72 ^°^C for 1 min and final elongation at 72 ^°^C for 5 min. Electrophoresis was performed on 1.5% agarose gel in 0.5X Tris-Borate EDTA (TBE) buffer. A 100 bp Plus DNA ladder (SinaClon) was used as a size marker. The DNA fragments were stained with Safe stain (SinaClon) and visualized under UV transilluminator. The primers used are presented in Table 1.

***Statistical analysis***

Data were analyzed using SPSS software (ver. 16). Multidrug-resistant (MDR) was defined as non-susceptible strains to at least one agent in three or more different antimicrobial categories and an MRSA is always considered MDR (19). Pearson’s Chi-Squared test or Fisher’s exact test where appropriate was used to determine significance. A *P*-value<0.05 was considered significant.

## Results

Of the 70 isolates, 40 (57.1%) were isolated from males, therefore 30 (42.9%) were isolated from females. Fifteen of the 70 isolates (21.4%) were from outpatients and 55 (78.6%) from inpatients admitted to different wards, including infectious diseases (n=15), emergency (n=9), women (n=7), intensive care unit (ICU, n=6), men (n=5), burn (n=4), coronary care unit (CCU, n=2), cardiology (n=2), digestive (n=2), dialysis, nervous, and pediatric (n=1, each) wards. The average age of patients was approximately 56 years. The age range was from 13 years old (one patient) to 92 years old (two patients) and most patients were between 60–69 years old (13 of the 70 patients, 18.6%). The strains were mostly isolated from urine (n=43, 61.4%), followed by blood (n=16, 22.9%), wound (n=7, 10%), tracheal secretions (n=2, 2.9%), cerebrospinal fluid, and catheter (n=1, 1.4%; each).

***Antibiotic susceptibility***

The most effective antibiotics were linezolid, vancomycin, and trimethoprim-sulfamethoxazole (above 90% sensitivity) and the least effective antibiotics were penicillin and erythromycin (4.3% and 47.1% sensitivity, respectively). Twelve isolates (17.1%) were resistant to cefoxitin and were identified as MRSA; thus, 58 isolates (82.9%) were methicillin-sensitive *S. aureus* (MSSA). Of the 12 MRSA isolates, 7 (58.3%) were isolated from the outpatients and 5 (41.7%) from the inpatients. Interestingly, antibiotic susceptibility of the strains isolated from the in-patients was higher than the outpatients and a significant association according to Fisher’s exact test was seen for susceptibility to tetracycline, cefoxitin, and ciprofloxacin between the outpatients and inpatients. The susceptibility rate of the isolates to 10 antibiotics is presented in Table 2.

The vancomycin MIC, which was determined by the E. test, ranged from 0.25 to 3 μg/ml for all isolates. Most isolates had a MIC of 1.5 μg/ml (27 isolates) followed by 17 isolates with a MIC of 2 μg/ml. Four out of the 70 isolates (5.7%) had vancomycin MIC of 3 µg/ml and were classified as vancomycin-intermediate *S. aureus *(VISA) based on CLSI 2019 (12). Three of the 4 VISA strains were MSSA; thus, one isolate was determined as MRSA. Two of the 4 strains were isolated from the blood and urine of the inpatients and 2 others from the urine of the outpatients. Of the 2 VISA isolates from the outpatients, one was only susceptible to linezolid (MRSA) and the other isolate was sensitive to all antibiotics except penicillin, erythromycin, ciprofloxacin, and vancomycin (MSSA). Of the 2 VISA isolates from the inpatients, one was sensitive to trimethoprim-sulfamethoxazole, linezolid, gentamicin, and cefoxitin (MSSA) and the other isolate was sensitive to all antibiotics except penicillin, erythromycin, and vancomycin (MSSA).

The MSSA isolates were more susceptible to the antibiotics than the MRSA isolates. The susceptibility rate of the 12 MRSA isolates to the antibiotics was 100% (n=12) for linezolid, followed by 91.7% (n=11) for vancomycin, 75% (n=9) for trimethoprim-sulfamethoxazole, 33.3% (n=4) for gentamicin, 25% (n=3) for tetracycline and clindamycin (each), 16.7% (n=2) for ciprofloxacin, and 8.3% (n=1) for erythromycin. All MRSA isolates were resistant to penicillin. The susceptibility rates of the 58 MSSA isolates were 100% (n=58) for linezolid and trimethoprim-sulfamethoxazole, 98.3% (n=57) for gentamicin, 94.8% (n=55) for vancomycin, 84.5% (n=49) for tetracycline, 81% (n=47) for ciprofloxacin, 79.3% (n=46) for clindamycin, 55.2% (n=32) for erythromycin, and 5.2% (n=3) for penicillin.

Twenty non-susceptibility patterns were identified among the 70 isolates. The patterns ranged from non-susceptibility only to one antibiotic to non-susceptibility against 9 of the 10 antibiotics. Of the 70 isolates, 27 isolates were non-susceptible to one antibiotic (38.6%), 30 were non-susceptible to 2 to 5 antibiotics (42.8%), and 10 were non-susceptible to 6 to 9 antibiotics (14.3%). The most frequently detected pattern was non-susceptibility only to penicillin (27/70, 38.6%) followed by penicillin and erythromycin combination (10/70, 14.3%). Three isolates were susceptible to all antibiotics.

Nearly 95.7% of all isolates (67/70) were non-susceptible to at least one antibiotic and 28 of the 70 isolates (40%) were MDR. The major patterns detected within the 58 MSSA isolates were non-susceptibility only to penicillin (n=27) followed by non-susceptibility to penicillin and erythromycin combination (n=10). Within the MRSA isolates, no pattern was dominant; however, non-susceptibility to six or more agents was more frequently seen in the MRSA isolates and the majority of the MSSA isolates were non-susceptible only to one or two antibiotics (Table 3).

***Distribution of integrons, mecA, SCCmec,*** ***and agr ***

PCR assay showed that 6 of the 70 isolates (8.6%) carried class 1 integron; classes 2 and 3 were not found. The major non-susceptibility pattern detected within the 64 integron-negative isolates was non-susceptibility only to penicillin (n=24) followed by non-susceptibility to penicillin and erythromycin combination (n=9). Within the 6 integron-positive isolates, no pattern was dominant and the identified patterns were non-susceptibility to penicillin (n=3), penicillin and erythromycin (n=1), penicillin and cefoxitin (n=1), and one isolate was non-susceptible to all antibiotics except linezolid and vancomycin (Table 3). 

The *mecA* gene was found in 8 of the 70 isolates (11.4%); therefore, of the 12 isolates which were detected as MRSA by the cefoxitin disk diffusion test, 4 isolates were *mecA*-negative. Of the 8 *mecA*-positive isolates, 2 isolates carried SCC*mec *III, 2 isolates SCC*mec *V, and 4 isolates were non-typeable. SCC*mec* types I, II, and IV were not found. Five of the 8 *mecA* -positive isolates were from outpatients and 3 isolates were from inpatients. 

Assays for detection of *agr* showed that 12 of the 70 isolates carried *agr* I (17.1%) and 21 isolates (30%) carried *agr* III. Types II and IV were not found. Of the four isolates with intermediate resistance to vancomycin, one isolate had *agr* I, and three isolates were non-typeable. The prevalence of both *agr* I and III was higher in the inpatients than the outpatients (10 of 12 *agr* I isolates (83.3%) and 19 of 21 *agr *III isolates (90.5%) were isolated from inpatients).

**Figure 1:**
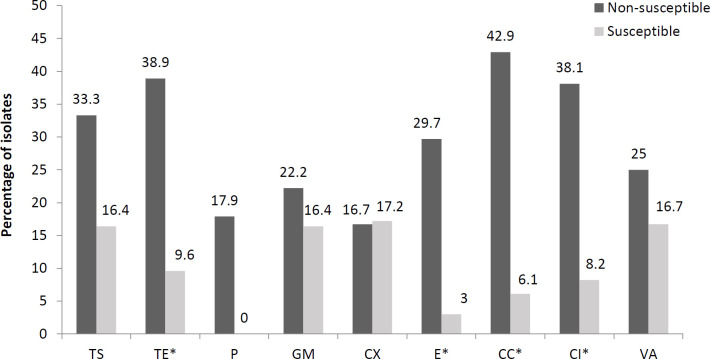
Prevalence of *agr* I in antibiotic-susceptible and -non-susceptible *Staphylococcus aureus* isolates. *: *P*-value<0.05. *P*-value was defined according to Fisher’s exact test. TS: trimethoprim-sulfamethoxazole; TE: tetracycline; P: penicillin; GM: gentamicin; CX: cefoxitin; E: erythromycin; CC: clindamycin; CI: ciprofloxacin; VA: vancomycin

**Figure 2:**
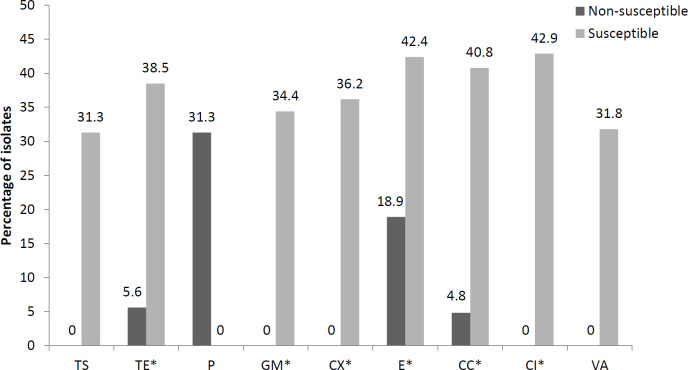
Prevalence of *agr* III in antibiotic-susceptible and -non-susceptible *Staphylococcus aureus *isolates. *: *P*-value<0.05. TS: trimethoprim-sulfamethoxazole; TE: tetracycline; P: penicillin; GM: gentamicin; CX: cefoxitin; E: erythromycin; CC: clindamycin; CI: ciprofloxacin; VA: vancomycin

In general, the prevalence of *agr* I was higher in the non-susceptible isolates than in the susceptible isolates (because all isolates were susceptible to linezolid, this drug was excluded). There was an association shown by Fisher’s test between resistance to tetracycline, erythromycin, clindamycin, and ciprofloxacin with the presence of *agr* I (*P*-value < 0.05). The prevalence of *agr* I in the MSSA isolates was slightly higher than in the MRSA isolates (Figure 1). Conversely, the prevalence of *agr *III in the susceptible isolates was higher than in the non-susceptible isolates, and there was a significant relationship between sensitivity to tetracycline, gentamicin, cefoxitin, erythromycin, clindamycin, and ciprofloxacin with the presence of *agr* III (*P*-value < 0.05). The *agr* III isolates were significantly susceptible to cefoxitin (MSSA), gentamicin, and ciprofloxacin, each and no MRSA isolates belonged to *agr *group III (Figure 2).

**Table 1:** Sequence of primers, annealing temperatures, and predicted size of PCR products

**Target gene**	**Primer sequence (** **5′-3′)**	**Amplification product (bp)**	**Annealing temperature (°C)**	**Ref.**
*mecA*	GTGAAGATATACCAAGTGATT/ ATGCGCTATAGATTGAAAGGAT	147	54	(15)
SCC*mec*I	GCTTTAAAGAGTGTCGTTACAGG/ GTTCTCTCATAGTATGACGTCC	613	62	(15)
SCC*mec* II	CGTTGAAGATGATGAAGCG/ CGAAATCAATGGTTAATGGACC	398	54	(15)
SCC*mec*III	CCATATTGTGTACGATGCG/ CCTTAGTTGTCGTAACAGATCG	280	54	(15)
SCC*mec* IV	GCCTTATTCGAAGAAACCG/ CTACTCTTCTGAAAAGCGTCG	776	56	(15)
SCC*mec* V	GAACATTGTTACTTAAATGAGC/ TGAAAGTTGTACCCTTGACACC	325	60	(15)
*agr I*	ATGCACATGGTGCACATGC/ GTCACAAGTACTATAAGCTGCGAT	440	61	(16)
*agr *II	ATGCACATGGTGCACATGC/ GTATTACTAATTGAAAAGTGCCATAGC	572	59	(16)
*agr *III	ATGCACATGGTGCACATGC/ CTGTTGAAAAAGTCAACTAAAAGCTC	406	57	(16)
*agr *IV	ATGCACATGGTGCACATGC/ CGATAATGCCGTAATACCCG	588	56	(16)
*intI1*	CAGTGGACATAAGCCTGTTC/ CCCGAGGCATAGACTGTA	160	54	(17)
*intI2*	CACGGATATGCGACAAAAAGGT/ GTAGCAAACGAGTGACGAAATG	789	60	(18)
*intI3*	GCCTCCGGCAGCGACTTTCAG/ ACGGATCTGCCAAACCTGACT	979	60	(18)

**Table 2:** Antibiotic susceptibility of 70 *Staphylococcus aureus* isolates from outpatients and inpatients

**Antibiotics ** ^a^	**Total ** **N= 70 (%)**	**Outpatients N= 15 (%)**	**Inpatients N= 55 (%)**
LZ	70 (100)	15 (100)	55 (100)
TS	67(95.7)	13 (86.7)	54 (98.2)
VA	66 (94.3)	13 (86.7)	53(96.4)
GM	61(87.1)	11 (73.3)	50 (90.9)
CX*	58 (82.9)	8 (53.3)	50 (90.9)
TE*	52 (74.3)	7 (46.7)	45 (81.8)
CC	49 (70)	7 (46.7)	42 (76.4)
CI*	49 (70)	6 (40)	43 (78.2)
E	33 (47.1)	4 (26.7)	29 (52.7)
P	3 (4.3)	0	3(5.5)

^a ^TS: trimethoprim-sulfamethoxazole; TE: tetracycline; P: penicillin; LZ: linezolid; GM: gentamicin; CX: cefoxitin; E: erythromycin; CC: clindamycin; CI: ciprofloxacin; VA: vancomycin

*: *P*-value<0.05 according to Fisher’s exact test

**Table 3:** Antibiotic non-susceptibility patterns of 70 *Staphylococcus aureus* isolates from clinical samples

**Non-susceptibility** ** pattern ** ^a^	**No. of isolates in**			
	**MRSA** ^b^ **N= 12 (%)**	**MSSA** ^c^ **N= 58 (%)**	**Integr** **o** **n I-positive isolates N= 6 (%)**	**Integron I-negative isolates** ** N= 64 (%)**
P	0	27(46.5)	3 (50)	24 (37.5)
P,CI	0	1(1.7)	0	1 (1.6)
P,E	0	10(17.2)	1(16.7)	9 (14.1)
TE,P	0	1(1.7)	0	1(1.6)
P,CX (MDR)^b^	1(8.3)	0	1 (16.7)	0
P,E,VA (MDR)	0	1 (1.7)	0	1(1.6)
TE,P,E (MDR)	0	1(1.7)	0	1(1.6)
P,GM,E (MDR)	0	1(1.7)	0	1(1.6)
P,E,CC (MDR)	0	3 (5.2)	0	3 (4.7)
P, E,CI,VA (MDR)	0	1 (1.7)	0	1(1.6)
P,E,CC,CI (MDR)	0	2 (3.4)	0	2 (3.1)
TE,P,CX,E (MDR)	1(8.3)	0	0	1(1.6)
P,GM,CX,E,CI (MDR)	1(8.3)	0	0	1(1.6)
TE,P,E,CC,CI (MDR)	0	6 (10.3)	0	6 (9.4)
TE,P,E,CC,CI,VA (MDR)	0	1 (1.7)	0	1(1.6)
P,GM,CX,E,CC,CI (MDR)	1(8.3)	0	0	1(1.6)
TE,P,CX,E,CC,CI (MDR)	2(16.7)	0	0	2(3.1)
TE,P,GM,CX,E,CC,CI (MDR)	3(25)	0	0	3(4.7)
TS,TE,P,GM,CX,E,CC,CI (MDR)	2(16.7)	0	1(16.7)	1(1.6)
TS,TE,P,GM,CX,E,CC,CI,VA (MDR)	1(8.3)	0	0	1(1.6)
All susceptible	0	3 (5.2)	0	3(4.7)

^a ^TS: trimethoprim-sulfamethoxazole; TE: tetracycline; P: penicillin; LZ: linezolid; GM: gentamicin; CX: cefoxitin; E: erythromycin; CC: clindamycin; CI: ciprofloxacin; VA: vancomycin

^b ^MDR: multidrug-resistant (non-susceptible strains to at least 1 agent in 3 or more antimicrobial categories and an MRSA is considered MDR (19)

## Discussion

Between December 2017 and May 2018, we isolated 70 *S. aureus* from different clinical specimens of patients in two referral hospitals of Sanandaj, western Iran. The strains were mostly isolated from the inpatients and the majority of patients were between 60–69 years old, which can be explained by the fact that *S. aureus* infections occur more frequently in patients with decreased immunity or underlying diseases such as the elderly as well as in those hospitalized. Prolonged duration of therapy, extensive use of antibiotics, underlying illness, surgical wounds or burns, and decreased immunity are the main risk factors for the acquisition of *S. aureus* infections (1).

In the present study, the most effective antibiotics for *S. aureus* isolates were linezolid, trimethoprim-sulfamethoxazole, and vancomycin, and the least effective were penicillin and erythromycin, which is almost similar to other reports from Iran (20-22), China (23), and Pakistan (24). However, the prevalence of MRSA in our study was lower than that in the above studies. In our study, 17.1% of the isolates were MRSA and 40% of the isolates were MDR; while in a study from Iran in 2015 (22), 78.4% and 80.5% of 139 *S. aureus* isolates were MDR and MRSA, respectively or in another study from Iran in 2016 (21), of 80 *S. aureus *isolates, all were MDR and 86.2% were MRSA. In Pakistan in 2014, MRSA prevalence was found to be 51.1% according to the cefoxitin test (24). In China in 2014, a prevalence of 59.9% was found for MRSA isolated from urogenital tract infection (23). This variation in the prevalence rates may be explained in part by different local antibiotic prescriptions, infection control programs, transmission of resistant isolates, and studied population (25). Interestingly, in our study, the non-susceptibility rates in the isolates from the outpatients were higher than those in the isolates from the inpatients, especially for tetracycline, cefoxitin, and ciprofloxacin, likely due to the excessive and/or inappropriate use of these antimicrobials in the community, ineffective contact precautions for MRSA and the lack of stringent antibiotic policies in the community (25). 

MRSA strains tend to be multi-resistant against many currently available antimicrobial drugs (1). Accordingly, the rates of resistance among our MRSA isolates were higher than those among the MSSA isolates and non-susceptibility to 6 or more antibiotics was more frequently seen in the MRSA isolates, while the majority of the MSSA isolates were non-susceptible only to one or two antibiotics. Out of the 12 isolates which were detected as MRSA by the cefoxitin test, 4 isolates were *mecA*-negative. Mechanism of methicillin resistance other than *mecA* has been reported and includes a novel *mecA* homolog, *mecC*. Resistance due to *mecC* is rare and cannot be detected by the tests detecting *mecA *(12).

Vancomycin is one of the most effective treatment options for *S. aureus* infections (20). There are few reports about the isolation of *S. aureus* with reduced susceptibility to vancomycin in Iran. In 2015, Mirzaee *et al.* found 11 (2.65%) strains with intermediate resistance to vancomycin among 415 clinical isolates of *S. aureus* (26). Furthermore, out of the 30 MRSA isolated from Tehran in 2017, 2 strains were vancomycin-resistant with MIC higher than 128 μg/ ml (27). In our study, out of the 70 isolates, 4 isolates (5.7%) with intermediate resistance to vancomycin were isolated from outpatient and hospitalized patients. Although the rate of vancomycin resistance in our study was low, it indicates the need for caution in the use of this antibiotic both in the community and in the hospital in Sanandaj. 

Many factors are responsible for resistance of bacteria to antimicrobials including presence of mobile genetic elements such as integrons (9). A low prevalence of class 1 integron was identified in our isolates (8.6%) and classes 2 and 3 were not found. However, a high prevalence rate of 72.6% for class 1 integron and 35.2% for class 2 integrons in *S. aureus* isolates from Iran was reported (22). Furthermore, in another study in Iran, class 1 and 2 integrons were found in 56.3% and 18.7% of the isolates, respectively (21). In China, a prevalence rate of 53% for class 1 integrase was reported (28). Class 1 integrons in *S. aureus* were significantly associated with resistance to certain antibiotics, including aminoglycosides, beta-lactams, trimethoprim, and chloramphenicol (21, 28). Although we could not characterize the content of integrons due to some limitations, the characterization of non-susceptibility patterns in the integron-positive and -negative isolates may partly suggest the gene content of the integrons in the isolates. 

*agr *typing showed that 30% and 17.1% of our isolates carried *agr* III and *agr* I, respectively. However, in several studies in Iran, such as those conducted. In Tehran in 2012 (29), in Isfahan and Shahrekord in 2019 (30), and in Tehran in 2017 (31)* agr *type I was the most dominant. Studies conducted in Korea in 2016 (32), in Turkey in 2017 (33), and in Pakistan in 2014 (24) indicated that *agr *I was the most predominant *S. aureus *type. This is more likely due to ecological and geographical differences. In our study, the *agr* III isolates showed more susceptibility to antibiotics than the *agr* I isolates, and *agr* III was not found in MRSA and the isolates non-susceptible to gentamicin and ciprofloxacin, each (*P*-value<0.05). The role of *agr* in antibiotic treatment is controversial. Data have demonstrated that the majority of MRSA in France belong to *agr* III (34). In Pakistan, *agr *III isolates showed higher antibiotic resistance than *agr *I isolates (24). In the study by Azimian *et al.* in Iran, most MRSA strains belonged to *agr *I and III, and most of MSSA strains belonged to *agr* II and IV (29). However, another study in Iran reported that most MRSA isolates belonged to *agr* I and none of them belonged to *agr *III; though, no significant difference was found regarding the presence of *agr *groups between MRSA and MSSA strains (35). Furthermore, a study conducted in Iran found a significant correlation between *agr *I and resistance to cefoxitin and erythromycin (*P*-value<0.05) (30).

## Coclusion

Our study suggests that for treatment of patients with *S. aureus *infections, trimethoprim-sulfamethoxazole, vancomycin, or linezolid would be suitable agents. Although the prevalence of *mecA* and integrons in our study was relatively low, the identification of such strains calls for serious health concerns. These data suggest that *agr* activity already known to affect the production of virulence factors may also affect the resistance of *S. aureus* to antibiotics. Further studies with larger sample sizes and determination of MIC for all tested antibiotics provide more data about the prevalence of MRSA isolates and the effect of *agr* typing on the treatment of *S. aureus* infections in western Iran. 

## Ethical Code

IR.MUK.REC.1396.11, Research Ethics Committee (REC), Faculty of Medicine, Kurdistan University of Medical Sciences
